# tsRNAs: Novel small molecules from cell function and regulatory mechanism to therapeutic targets

**DOI:** 10.1111/cpr.12977

**Published:** 2021-01-28

**Authors:** Tingyu Zong, Yanyan Yang, Hui Zhao, Lin Li, Meixin Liu, Xiuxiu Fu, Guozhang Tang, Hong Zhou, Lynn Htet Htet Aung, Peifeng Li, Jianxun Wang, Zhibin Wang, Tao Yu

**Affiliations:** ^1^ Department of Cardiac Ultrasound The Affiliated Hospital of Qingdao University Qingdao China; ^2^ Department of Immunology School of Basic Medicine Qingdao University Qingdao China; ^3^ Department of Radiology The Affiliated Hospital of Qingdao University Qingdao China; ^4^ Department of Vascular surgery Qingdao Hiser Medical Center Qingdao China; ^5^ Institute for Translational Medicine The Affiliated Hospital of Qingdao University Qingdao China

**Keywords:** biomarker, cell proliferation, clinical, therapeutic target, tRNA‐derived small RNAs

## Abstract

tsRNAs are small fragments of RNAs with specific lengths that are generated by particular ribonucleases, such as dicer and angiogenin (ANG), clipping on the rings of transfer RNAs (tRNAs) in specific cells and tissues under specific conditions. Depending on where the splicing site is, tsRNAs can be segmented into two main types, tRNA‐derived stress‐induced RNAs (tiRNAs) and tRNA‐derived fragments (tRFs). Many studies have shown that tsRNAs are functional molecules, not the random degradative products of tRNAs. Notably, due to their regulatory mechanism in regulating mRNA stability, transcription, ribosomal RNA (rRNA) synthesis and RNA reverse transcription, tsRNAs are significantly involved in the cell function, such as cell proliferation, migration, cycle and apoptosis, as well as the occurrence and development of a variety of diseases. In addition, tsRNAs may represent a new generation of clinical biomarkers or therapeutic targets because of their stable structures, high conservation and widely distribution, particularly in the peripheral tissues, bodily fluids and exosomes. In this review, we describe the generation, function and mechanism of tsRNAs and illustrate the current research progress of tsRNAs in various diseases, highlight their potentials as biomarkers and therapeutic targets in clinical application. Although our understanding of tsRNAs is still in infancy, the application prospects shown in this field deserve further exploration.

## Graphical abstract

tsRNAs are generated under stress condition which are functional molecules. Due to their regulatory mechanism in regulating mRNA stability, transcription, ribosomal RNA (rRNA) synthesis and RNA reverse transcription, tsRNAs are significantly involved in the cellular function, including cell proliferation, migration, cell cycle and apoptosis, as well as the occurrence and development of a variety of diseases.

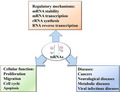

## 1 INTRODUCTION

In 1958, Francis Crick proposed the central dogma of molecular biology: that genomic DNA in the nucleus is transcribed into messenger RNA (mRNA), which is then translated into proteins when it enters the cytoplasm. It is traditionally believed that, at the initial stage of translation, amino acids and their homologous tRNAs undergo aminoacylation through the action of aminoacyl‐tRNA synthetase (AARS) to form an aminoacyl‐tRNA complex (aa‐tRNA).^1^, ^2^ Then, aa‐tRNA specifically recognizes the codon on the mRNA sequence, binds to the rRNA‐mRNA complex and connects the corresponding amino acid to the polypeptide chain under the action of rRNA so that the polypeptide chain keeps extending.^3^ However, protein‐coding genes and related transcripts in different species account for only about 2% of the entire human genome.^4^ The vast majority of the remaining sequences do not encode proteins, suggesting that non‐coding RNAs (ncRNAs) may dominate the eukaryotic transcriptome.^5^ ncRNAs include long ncRNAs (lncRNAs) and small non‐coding RNAs, divided by the length of 200 nucleotides (nts). There are several types of regulatory small non‐coding RNAs, including microRNAs (miRNAs), piwi‐interacting RNAs (piRNAs) and small interfering RNAs (siRNAs).^6^, ^7^, ^8^, ^9^, ^10^, ^11^, ^12^ These ncRNAs have been reported to be involved in a number of biological activities, such as cell proliferation and migration, the cell cycle, apoptosis and autophagy.^13^, ^14^, ^15^ Of note, they are closely related to various diseases, such as cancer, cardiovascular diseases, nervous system diseases and inflammation.^13^, ^16^, ^17^, ^18^, ^19^, ^20^, ^21^, ^22^ In recent years, with the rapid advancement of sequencing technology, a cluster of new small ncRNAs, namely, tsRNAs, has been discovered.^23^

tsRNAs are not randomly degraded fragments of tRNAs but are rather a class of abundant and novel RNAs with precise sequence structures as well as specific biological functions.^24^ These new small RNAs, as members of the ncRNA family, are not yet fully understood. Based on the present understanding, tsRNAs undergo special cleavage and are then modified at the 5′‐ and 3′‐ends to give them the property of stability.^25^ tsRNAs were reported to be implicated in several mechanisms, such as regulating mRNA stability, translation, rRNA synthesis and RNA reverse transcription. Accordingly, tsRNAs participate in regulating biological functions through these mechanisms, including cell proliferation, migration, cycle and apoptosis. Similarly, many studies have shown that tsRNAs have tissue specificity and temporal specificity and are expressed abnormally in numerous diseases, such as cancer, neurological diseases, metabolic diseases and viral infectious diseases.^26^, ^27^ More recently, the essential roles of tsRNAs in functions and mechanisms of various diseases were investigated.

In this review, we generalize the biological features, biogenesis, biology function and regulatory mechanisms of tsRNAs and illustrate the critical involvement of tsRNAs in the progression and regulation of cancer and other diseases. Finally, we discuss the potential of tsRNAs for new diagnostic biomarkers and clinical therapeutic strategies, despite the limitations of current research.

## 2 THE IDENTIFICATION OF TSRNAS

The origin of tsRNAs dates back to the late 1970s, when they were originally discovered in tumour cells.^28^, ^29^ However, they were merely identified as random degradation products from tRNA sources and had not attracted intense attention in the field of scientific research. Owing to the development of science and technology, tsRNAs were sequentially discovered in the human,^30^ protists,^31^ bacteria^32^ and virus.^33^ Some tsRNAs are evolutionarily conservative, despite their differential evolution between species.^34^ In addition, they are not only widely expressed, but also have abundant sources. Because a single amino acid has many different tRNA acceptors, and an anti‐codon corresponds to many different tRNA acceptors, various types of tsRNAs can come from diverse tRNA sources. Moreover, accumulating evidence suggests that tsRNAs have special methylation modification and terminal modification, including cyclic phosphorylation modification, 5‐OH modification and aminoacyl modification,^35^ which modification occurs may be depended on the type of tsRNAs. In addition, the production of tsRNAs could be affected and activated by phosphate starvation,^36^ hypoxia,^37^ oxidative damage^38^ and other malignant conditions. However, the level of tsRNAs rarely changes under normal conditions.^39^ tsRNAs are considered to be functional molecules and not as by‐products of non‐functional degradation.^39^ Thus, tsRNAs are not the tRNA random degradation products,^23^ but a novel cluster of small molecular RNAs splits from tRNAs, with inherent conservation, high expression and good stability.

## 3 THE BIOGENESIS OF TSRNAS: TSRNAS ARE USUALLY DIVIDED INTO TWO CATEGORIES WITH SPECIAL SPLICING PATTERNS

**FIGURE 1:**
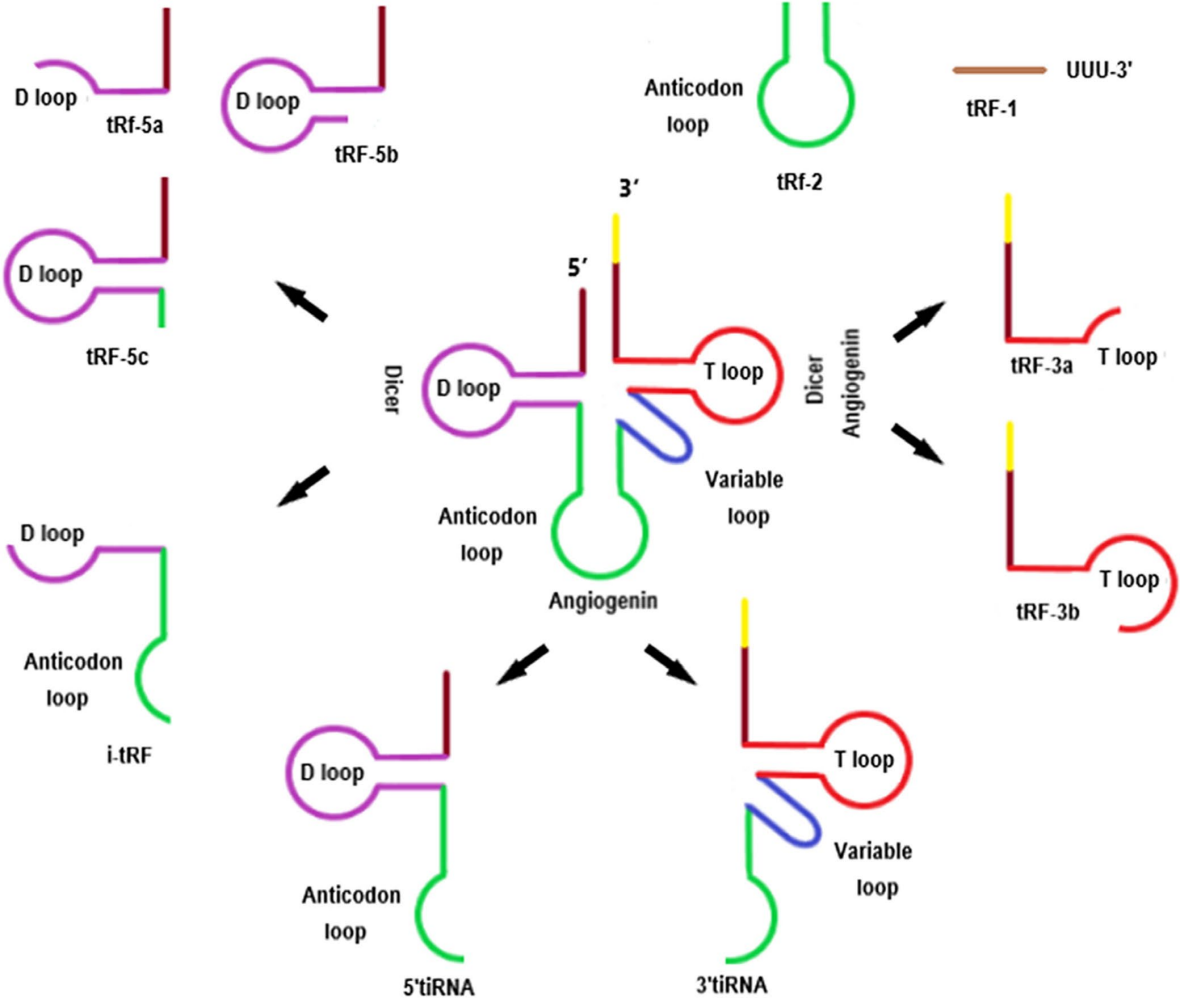
The biogenesis of tsRNAs. tsRNAs can be generally classified into the following two categories according to the cleavage sites in tRNAs and lengths: tiRNAs and tRFs. tiRNAs are classed by their source sequences from the 5′‐ or 3′‐end of tRNA cleaved by ANG. tRFs are classified into three major types depending on the size and location of the source: tRF‐5, tRF‐3 and tRF‐1. The current generation mechanisms of 2‐tRF and i‐tRF are still not completely clear

The earliest of tRNA cleavage was observed in *Escherichia coli* (*E coli*) as a response to bacteriophage infection.^40^ To better understand the biological production process of tsRNAs, it is necessary to first understand the structural characteristics of tRNAs. tRNA molecules are produced in the form of precursors and then go through a series of maturation events, including RNase P trimming the 5′ beginning end, RNase Z taking out the 3′ trailer, removing the introns and adding the 3′‐terminal CCA sequence catalysed by CCA‐adding enzymes.^41^ Mature tRNAs have cloverleaf secondary structures with lengths of approximately 70‐90 nts, and each tRNA includes the amino acid acceptor arm, the D‐loop, the anti‐codon loop, the variable loop and the TψC loop (also called the T‐loop).^42^ Furthermore, tRNAs have tertiary structures that look like an ‘L’, also called an L‐shaped structure. tsRNAs usually can be divided into the following two subtypes depending on the cleavage sites in tRNAs and lengths: tiRNAs and tRFs.^20^ tiRNAs are 28‐36 nts long, generated by particular ribonucleases cleaving the anti‐codon loops of mature tRNAs,^20^, ^43^ while tRFs, which are 15‐32 nts in length, are produced by clipping the ends of precursor or mature tRNAs (Table 1). Accordingly, the two categories of tsRNAs are produced under different conditions and in different ways (Figure 1).

**TABLE 1:** The classification of tsRNAs

tsRNAs	Category	Length (nts)	Nucleases	Cleavage sites	REFERENCES
tiRNAs	5′‐tiRNAs	28‐36	ANG (mammals) RNY1 (yeast)	The anti‐codon loop of mature tRNA	43, 44
	3′‐tiRNAs				
tRFs	tRF‐5a	14‐16	Dicer (mice, *Drosophila* and *Schizosaccharomyces. pombe*)	D‐loop or the stem region between D‐loop and anti‐codon loop of mature tRNAs	48, 49
	tRF‐5b	22‐24			
	tRF‐5c	28‐30			
	tRF‐3a	~18	ANG (human)	TΨC loop of mature tRNAs	48, 50, 51
	tRF‐3b	~22			
	tRF‐1	15‐ 32	RNase Z or ELAC2 (human)	Near the 3′‐ends of pre‐tRNAs	23, 51, 52, 53
	tRF‐2	15‐ 32	NA	NA	54, 55
	i‐tRF	15‐ 32	NA	NA	55

Abbreviation: NA, not acquired.

### 3.1 The biogenesis of tiRNAs

The biogenesis processes of tiRNAs usually occur when cells experience stress conditions.^29^ ANG is a member of the ribonuclease A superfamily, whose major function is promoting rRNA transcription and cell growth under growth conditions in the mammalian cell nucleus; however, ANG is mobilized to the cytosol to promote tRNA cleavage under stress conditions.^44^, ^45^, ^46^, ^47^ As a member of Ribonuclease T2 family, RNA, Ro60‐associated Y1 (RNY1) can meditate the biogenesis processes of tiRNAs in yeast.^43^ The ribonucleases above can cleave in the anti‐codon loop of mature tRNA, forming two subtypes, called the 5′‐tiRNAs and 3′‐tiRNAs.^43^ 5′‐tiRNAs are found from the 5′‐ends to the cleavage sites, while 3′‐tiRNAs are found from the cleavage sites to the 3′‐ends.^47^ These fragments are usually 28‐36 nts long.

### 3.2 The biogenesis of tRFs

The biogenesis of tRFs occurs under similar conditions as that of tiRNAs, but the fragments are smaller with lengths of 15‐32 nts.^48^, ^49^ tRFs have precise cleavage sites at the tRNA terminus, mainly determined by ribonucleases. According to the type of ribonuclease, tRFs contain type I tRFs and type II tRFs. From these, type I tRFs require Dicer to participate in the generation process, while instead, the 5′end of type II tsRNA is produced by the cleavage of RNaseZ, and RNA polymerase III is required to terminate transcription at the 3′end.^30^ But the specific cleavage mechanisms are still under investigation.^26^, ^30^ Apart from above classification, tRFs can also be generally divided into three major types based on cleavage sites on the precursor or mature tRNA transcripts:tRF‐5,tRF‐3 and tRF‐1. The first type is tRF‐5, which is 14‐30 nts long and is produced by cutting the D‐loop or the site between the D‐loop and anti‐codon loop of the mature tRNA transcript. This type is further divided into three subtypes of tRF‐5 with different specific lengths; for example, tRF‐5a has a length of 14‐16 nts, tRF‐5b is 22‐24 nts, and tRF‐5c is 28‐30 nts.^48^, ^49^ tRF‐3 has two subclasses: tRF‐3a and tRF‐3b, produced by ANG as well as other ribonucleases of the Ribonuclease A superfamily cutting the T‐loop of mature tRNAs. They are ~18 or ~22 nts in length, which are smaller than tRF‐5.^48^, ^50^, ^51^ tRF‐1 is generated from 3′‐trailer fragment during the pre‐tRNA maturation process, and the 5′‐ends are cleaved by RNase Z or ELAC2, and the 3′‐ends are transcriptional termination signals of RNA pol III (UUUUU, UUCUU, GUCUU or AUCUU). Therefore, the length of tRF‐1 is very different.^23^, ^52^, ^53^ Moreover, there are other types of tRFs, for example, tRF‐2 and i‐tRF.^54^, ^55^ However, the exact mechanism is not entirely clear. The specific subcellular localization of tRFs is also important for their biogenesis; for example, tRF‐5 usually exists in the nucleus, while tRF‐3 and tRF‐1 are usually in the cytoplasm.^48^, ^49^ In addition, these fragments have 5′ phosphates and 3′ hydroxyls similar to the structure and size of miRNA, so that they have received attention recently.

### 3.3 The relationship between tRNA modifications and tsRNA biogenesis

It is known to be more than 170 kinds of RNA modifications so far, of them, 93 are existed in tRNAs with different frequency and distribution depending on organisms or tRNA species.^56^ Recently, tRNA modification abnormalities were found to not only influence the stability and function of tRNAs, but also affect the biogenesis of tsRNAs. Recent study showed that NOP2/Sun RNA methyltransferase 2 (NSun2) and DNA methyltransferase‐2 (Dnmt2) are both methyltransferases which can methylate C5 at cytosine residues (called m5C) to sustain tRNA stability and functions.^57^ By disturbing these two methyltransferases, NSun2 and Dnmt2, cytosine‐C5 tRNA methylation was deficient in the mice, which resulted in reduced tRNA stability and decrease in protein synthesis.^24^, ^25^ Except for the influence of tRNA, reduction in stability due to deficiency of m5C mediated by Dnmt2 could also result in a significant increase in tiRNAs derived from the cleavage tRNAs of ANG.^58^, ^59^ Deletion of Dnmt2 would induce the deletion of m5C at C38 on tRNA, which finally promote the shearing of tRNA, leading to the increase and accumulation of tsRNAs.^57^ On the contrary, upregulation of m5C modifications decreased the levels of tiRNAs by suppressing the cleavage anti‐codon loop of tRNAs by ANG.^60^ Moreover, Alkbh1 and ALKBH3 were reported as 1‐methyl adenosine (m1A) demethylases of tRNA. m1A demethylated tRNA is more sensitive to angiogenin (ANG) cleavage, which can reduce the stability of tRNA and increase tRNA cleavage, and finally produce tsRNAs in the anti‐codon region of tRNA.^61^, ^62^ There is another study found that the lack of the pseudouridine synthase 7 (PUS7) can also interfere with different types of tRFs, and the pseudouridylation (Ψ) regulates the levels of multiple tRFs in stress‐induced situations or specific cell types.^63^ In addition, studies have shown that certain modifications may promote the activity of RNAases. For example, in order to prevent the population from being infected by bacteriophages, bacteria will specifically cut their own tRNAs in a suicidal manner.^64^ Similarly, in order to avoid the proliferation of a non‐self yeast species, the yeast *Kluyveromyces lactis* uses endonuclease to cut the anti‐codons of various *Saccharomyces cerevisiae* tRNAs.^65^ The above two cases indicate that the modification of tRNA anti‐codon at the wobble uridine (U34) position, namely 5‐methoxycarbonyl‐methyl‐2‐thiouridine (mcm5s2U), is necessary for the enzyme protein tRNase to cleave tRNA. This modification promotes the activity of tRNase, but the defect of mcm5s2U at position U34 inhibits the activity of tRNase and the cleavage of tRNA.^66^, ^67^ The production of tRNAs is closely related to the activity of RNases. Although these studies do not clearly explain the molecular mechanism of the interaction between RNases and tRNA modifications, they do prove that tRNA modifications can affect the cleavage of tRNAs, which lead to the production of tRNAs. Collectively, the above studies show that abnormal tRNA modifications can cause self‐splicing, which supports the view that specific tRNA modifications can affect tRNA cleavage; on the other hand, it suggests that the production of tsRNAs is a coordinated process when the body is stimulated. Then, this feature also provides evidence for tsRNAs as diagnostic markers; that is, when the body is attacked by pathogenic factors, the body may cooperate and undergo programmed tRNA cleavage to produce tsRNAs as markers for disease diagnosis.

## 4 THE REGULATORY MECHANISMS OF TSRNA

**FIGURE 2:**
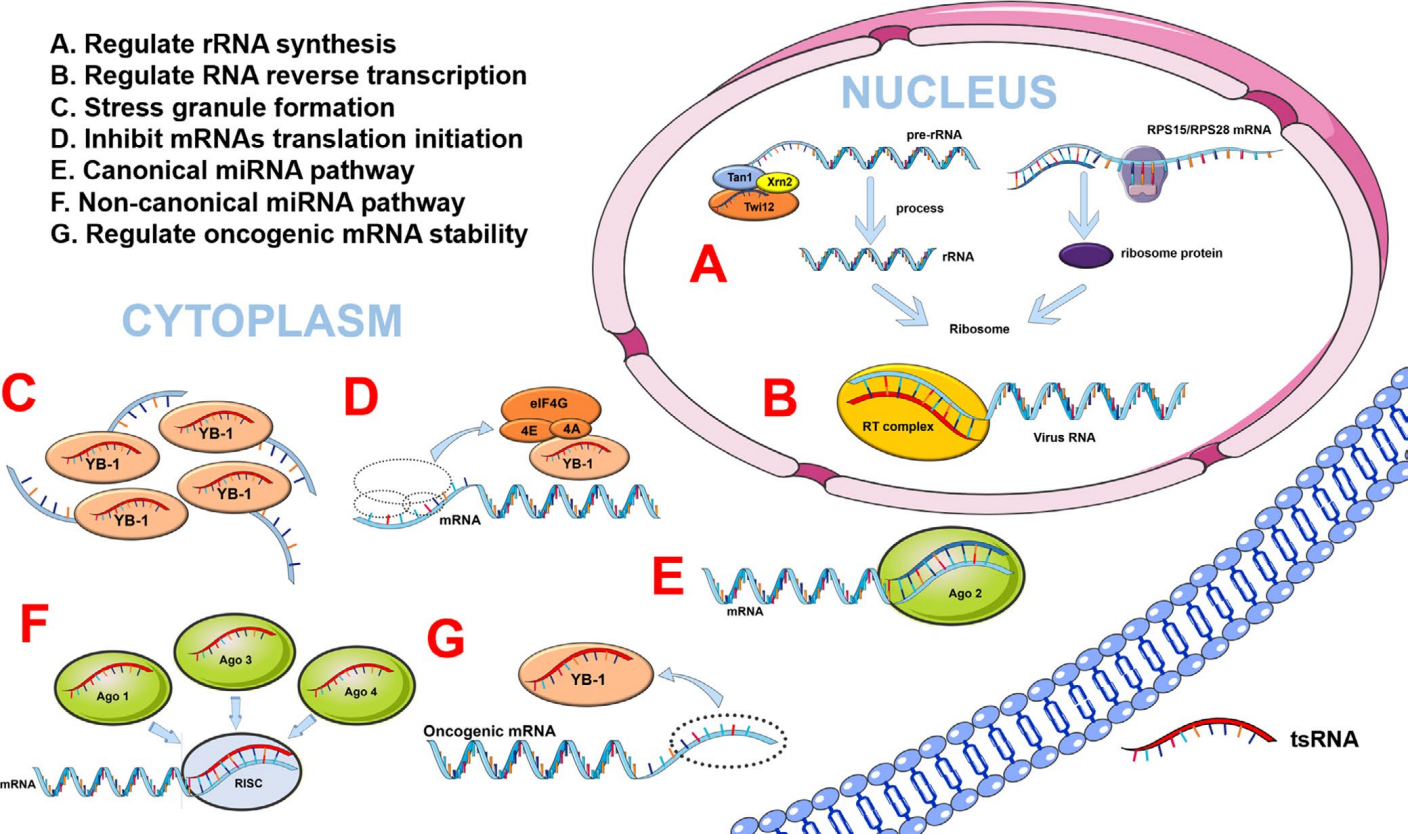
The regulatory mechanisms of tsRNAs. tsRNAs are involved in a variety of biological processes: (A) Regulate rRNA synthesis by acting as a part of the pre‐rRNA splicing complex; (B) Regulate the reverse transcription process of some viral RNAs; (C) Response to the stress by triggering the formation of stress granules; (D) inhibit mRNA translation initiation by connecting with the translation initiation complex; (E) Regulate mRNA stability in canonical miRNA pathway; (F) Regulate mRNA stability in non‐canonical miRNA pathway; (G) Regulate oncogenic mRNA stability. miRNA, microRNA; RISC, ‐induced silencing complex; rRNA, ribosome RNA; RT, reverse transcription; YB‐1, Y‐box binding protein 1

It is known that tsRNAs are specific fragments derived from tRNAs cleaved by ribonucleases, but they are not the products of random degradation. Indeed, studies have successfully clarified that tsRNAs are small non‐coding RNAs with regulatory functions, such as acting in mRNA binding (similar to miRNA) or as protein ‘sponges,’ regulating mRNA stability, inhibiting translation initiation and regulating ribosome biogenesis and RNA reverse transcription (Figure 2).

### 4.1 Regulating mRNA stability

tRFs are similar to miRNAs in structure and size, and, in particular, tRF‐3s derived from tRNA^Leu^ and tRNA^Lys^ are the same as the 3′‐end sequences of miR‐1280 and miR‐1274a/b, respectively.^68^, ^69^, ^70^ Additionally, miRNAs are a class of small non‐coding RNAs that regulate the combination of the miRNA‐induced silencing complex (miRISC) with the 3′ untranslated region (3′UTR) of partially complementary sites in target genes to regulate mRNA stability.^71^ Similarly, in a recent study, tRFs were found to have miRNA‐like biological functions. For example, 3‐tRF (also called CU1276) was a DICER1‐dependent tRNA^Gly‐GCC^‐derived tRNA fragment expressed in mature B lymphocytes to modulate proliferation and the DNA damage response.^72^ miRNA‐like functions of tRFs in *Drosophila* have also been verified: tRFs suppressed mRNA translation preferentially by binding their 5′‐ and 3′‐ends to conserved regions of 3′‐UTRs in mRNAs, and most tsRNAs are conserved, imminent and plentiful in *Drosophila* according to an analysis of 495 common small RNA libraries.^73^ Although most tRFs regulate the expression of target genes in a miRNA‐like way, recent studies showed that tRF‐3 played a role in inhibiting gene expression in a Dicer‐independent manner.^25^ In human HEK293 cells, tRFs were associated with Argonautes (Ago) 1, 3 and 4, but not with Ago 2, indicating that tRFs might play an important role in RNA silencing.^48^ Additionally, a novel class of tRFs has been discovered recently, displacing the 3′‐UTRs of YBX1 in breast cancer cells to suppress the stability of multiple oncogenic transcripts.^54^ However, the mechanism by which endogenous tRFs regulate target mRNA is still unclear, and further studies are pending to elucidate the regulatory network of tRFs.

### 4.2 tsRNAs regulate translation procedure

In addition to regulating mRNA stability by binding mRNA, tsRNAs also inhibit translation initiation by connecting with the translation initiation complex.^46^ ANG cleaved tRNAs within the anti‐codon loop of tRNAs to generate tiRNAs, and the transfection of natural or synthetic 5′tiRNAs triggered the formation of stress granules (SGs) assembled independently by phospho‐eIF2α, which were essential constituent parts of the stress response programme.^45^ 5′tiRNAs derived from tRNA^Ala^ and tRNA^Cys^ displaced eIF4F from the isolated m (7) G cap, but not eIF4E:4EBP1, to inhibit mRNA translation in the translation initiation complex and activate the cell‐protective effects in response to stress.^43^, ^46^ These discoveries indicated that the purpose of tiRNA production was to regulate translational procedures under stress stimulation instead of decreasing mature tRNA levels of encoding mRNAs. Moreover, SG‐dependent tiRNAs added oligoguanine motifs at their 5′‐ends to assemble G‐quadruplex‐like structures and bound to the translational silencer YB‐1 to inhibit translation initiation.^46^, ^74^, ^75^ Similarly, the inactivation of pseudouridine synthase 7 (PUS7), the pseudouridylation (Ψ) ‘writer,’ impaired tRF‐mediated translation regulation in embryonic stem cells, leading to translation inhibition.^63^ Recently, studies have found that there are two different ways in tsRNA‐mediated translation inhibition, namely AGO‐dependent and AGO‐independent methods.^76^ In the AGO‐dependent manner, AGO2 is indispensable for tsRNA‐mediated translational inhibition. tsRNA inhibits downstream target mRNA translation through antisense pairing. In other words, tsRNAs preferentially bind to AGO, and then, the 7‐mer motifs in tsRNA target conserved sites in mRNA and perform antisense matching, and finally the translation activity of targeted mRNA decreases.^76^ The tsRNA‐mediated AGO‐independent way is to influence the structure of mRNA or rRNAs and not rely on AGO protein. For example, 5′tsRNA‐Val does not target complementary binding to mRNA, but binds to the small ribosomal subunit (30S) 16S rRNA in the translation initiation complex, and then replaces the mRNA to inhibit the translation process.^76^, ^77^ In addition, ANG‐mediated tiRNAs reregulated protein translation, conserved energy for synthesis and metabolism, and maintained cell survival, which elucidated a novel mechanism for regulating cell growth and survival.^78^ This information enriches the diversity of the mechanisms of translation regulation through tsRNAs and proposes their potential as novel biomarkers for clinical testing or new strategies for disease therapy.

### 4.3 tsRNAs participate in regulating rRNA synthesis

Ribosomes decode the genetic information carried on mRNAs into a polypeptides chain, while ribosome biogenesis, from the transcription of pre‐rRNAs to the assembly of ribosomes, is regulated coordinately.^79^ Recent research has started to show that tsRNAs participate in the process of rRNA synthesis by acting as a part of the pre‐rRNA splicing complex (TXT) in protozoa (Tetrahymena).^80^ TXT is a complex with many components that contains 3‐tRF specifically binding with the Twi12 protein and exonuclease Xrn2. Upon binding, Twi12 stabilizes and localizes Xrn2 to stimulate the exonuclease, which cleaves the precursor rRNAs to regulate rRNA synthesis.^81^ Another piece of evidence showed that tRNA^Leu^‐derived tRF‐3 enhances ribosomal protein translation by binding to RPS28 and RPS15 and inducing apoptosis in splitting cells in vitro as well as in vivo.^82^ Even though this finding enriches the functional diversity of tsRNAs, current investigations regarding their function are still limited, and more research is needed to understand the mechanisms comprehensively.

### 4.4 tsRNAs regulate RNA reverse transcription

tsRNAs can regulate the reverse transcription process of some viral RNAs. On the one hand, a study found that 3′‐tRF has high expression levels in human immunodeficiency virus (HIV). HIV RNA was first hybridized with the 3′‐end of human tRNA^Lys^ to form a heterozygous double‐stranded RNA, which was then reversed‐transcribed into cDNA through its reverse transcriptase. The tRNA^Lys^ ‐derived 3′‐tRFs were able to promote the inhibition of HIV by binding AGO2 and Dicer.^33^ On the other hand, tsRNAs work by inhibiting viral reverse transcription. tRF‐3019 in host cells can start reverse transcription and promote self‐reproduction of the virus because tRF‐3019 exhibits a sequence identical to that of the primer binding site of HTLV‐1.^83^ Respiratory syncytial virus (RSV) infection of host cells produced more ANG‐mediated tiRNAs. These tiRNAs can act as primers for RSV by initiating and promoting reverse transcription,^51^, ^84^ thus improving RSV infection efficiency and reproductive capacity. The above studies indicate that tsRNAs are involved in the reverse transcription process of viruses, suggesting that tsRNAs may be the new targets for controlling viral infection.

### 4.5 Other potential regulatory mechanisms

Additionally, tsRNAs also participate in other potential functional mechanisms. For example, they could regulate cell proliferation and migration by inhibiting the expression of target genes.^85^ They were proven to participate in RNA transcription and DNA‐specific binding of proximal promoter of RNA polymerase II. Encouragingly, tsRNAs enveloped in exosomes were proved to be involved in communication signalling among cells.^86^ Another study showed patrilineal metabolic diseases may be passed on to offspring through spermatozoa, and this epigenetic inheritance was regulated by tsRNAs.^87^ Moreover, tsRNAs serve as important roles in immune response mediated by regulating T‐cell activation.^88^

As mentioned above, tsRNAs were reported to be implicated in several mechanisms, such as regulating mRNA stability, translation, rRNA synthesis and RNA reverse transcription. In the course of disease occurrence and development, the above mechanisms were more or less involved. Therefore, we have reasons to believe that tsRNAs may be used as a therapeutic method to inhibit the development and deterioration of diseases by regulating the expression of disease signalling pathways or their related genes.

## 5 tsRNAS IN HUMAN DISEASES

The discovery of tsRNAs is a recent breakthrough. The biological function and the regulatory mechanisms of different tsRNAs have been described above, along with these mechanisms corresponding to their roles in diseases. At present, a large amount of research shows that tsRNAs may play roles in the occurrence and development of various types of diseases, such as cancer, metabolic diseases, neurological diseases and viral infection diseases. Next, we will gradually explore the mechanisms of tsRNAs in diseases and potential therapeutic targets from the perspective of different diseases (Table 2).

**TABLE 2:** The function and mechanisms of tsRNAs in various diseases

Disease	tsRNAs	Expression	Target	Cell function	Disease model	Ref.
Colorectal cancer	tRF/miR‐1280	Downregulated	JAG2	Suppress CSC phenotypes and reduce cell proliferation	Athymic nude mice model of colorectal cancer xenograft with HCT116 cell	70
Breast cancer	5′‐tiRNAVal	Downregulated	FZD3	Reduce cell proliferation, migration and invasion	NA	85
Gastric cancer	tiRNA‐5034‐GluTTC‐2	Downregulated	NA	Suppress cell proliferation and invasion	NA	89
Lung cancer/ leukaemia	ts‐3676 ts‐4521	Downregulated	Piwil2	Suppress cell proliferation	NA	114
Prostate cancer	tRF‐1001	Upregulated	NA	Promote cell viability and cell proliferation	NA	23
Metabolic disease	tRFGly tRF‐Pro	Upregulated	NA	Influence the offspring cognition and hippocampal neurogenesis	Mice exercise model with a running wheel	93
Metabolic disease	tRFGly‐GCC	Upregulated	MERVL	Repress MERVL target genes in 2‐cell embryos	Mice fed with low protein diet	94
Metabolic disease	tiRNAs	Upregulated	NA	Affect metabolic gene expression through embryo to adulthood in the islet of F1 offspring	Paternal high‐fat diet (HFD) mouse models	87
Neurological diseases	3′tiRNA‐Gly‐CCC	Upregulated	NSun2 (upstream)	Reduce cell size and increased apoptosis of neurons	NSun2‐deficient mice	97
Spinal cord injury	tiRNA‐Gly‐GCC‐001	Upregulated	BDNF	Reduce cell survival under oxidative stress	Mice underwent laminectomy and contusion	103
RSV infection	tRF5‐ GluCTC	Upregulated	APOER2	Promote RSV replication in cells	NA	84
Spotted fever group rickettsioses	tRF5‐ValGTG	Upregulated	NA	Regulate endothelial cell proliferation, barrier function, autophagy and apoptosis	C3H/HeN mice infected with R. conorii	105

Abbreviation: NA, not acquired.

### 5.1 tsRNAs in cancer

**FIGURE 3:**
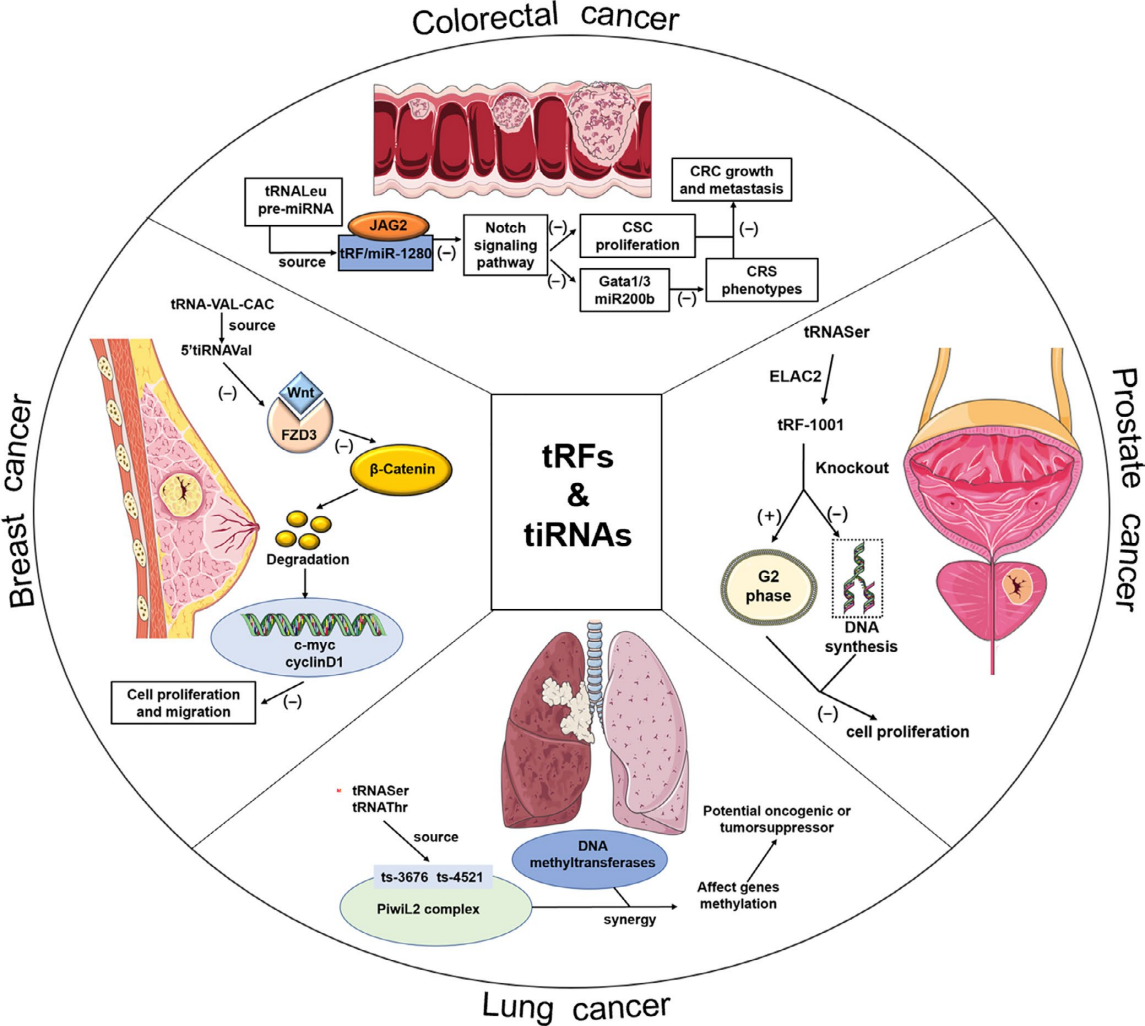
The role of tsRNA in cancers. tsRNAs are dysregulated in cancers indicating their contribution to various aspects of cancer progression (see text for details)

Cancer is a group of diseases characterized by abnormal cell proliferation and metastasis of cells, and specific biomarkers for diagnosis and treatment are currently unsatisfied. Of note, abnormal cell proliferation is the most important character of cancer. Interestingly, recent studies found that tsRNAs play significant roles in tumorigenesis either by promoting cell proliferation, migration and cell cycle progression or by inhibiting cancer progression^55^ (Figure 3). For example, colorectal and gastric cancers are both malignant tumours of the digestive system. In colorectal cancer (CRC), tRF/miR‐1280, derived from tRNA^Leu^ and pre‐miRNA, was implicated in combination with a direct target, Notch ligand jagged 2 (JAG2).^70^ Upon combination, Notch signalling pathways were suppressed, leading to inhibition of the cell proliferation of CRC. In particular, the inactivation of Notch signalling suppressed CSC phenotypes mediated by tRF/miR‐1280, accompanied by direct suppression of the Gata1/3 and miR200b genes.^70^ Moreover, 5′‐tiRNA^Val^ had a sensitivity of 90.0% and specificity of 62.7% and tiRNA‐5034‐GluTTC‐2 had a sensitivity of 84.7% and specificity of 92.8% in gastric cancer patients compared with healthy controls, which suggested that tiRNAs may be potential diagnostic biomarkers.^85^, ^89^ Moreover, a study proved for the first time that by binding with its direct target (FZD3), 5′‐tiRNA^Val^ could inhibit the Wnt/β‐catenin signalling pathway to suppress the cell proliferation and metastasis of breast cancer.^85^ Additionally, in prostate cancer, tsRNAs are able to inhibit cell proliferation by regulating the cell cycle.^23^ tRF‐1001, derived from tRNA^Ser^, is generated by the tRNA 3‐endonuclease ELAC2, a prostate cancer susceptibility gene. siRNA‐mediated silencing of the expression of tRF‐1001 suppressed cell proliferation by arresting cancer cells at the G2 phase and inhibiting DNA synthesis.^23^ The cell proliferation and cells accumulating in the G2 phase induced by si‐tRF1001 could also be rescued by co‐transfection of a synthetic 2‐O‐methyl tRF‐1001 oligoribonucleotide.^23^ As reported, breast cancer anti‐oestrogen resistance 3 (BCAR3) is essential for the development of ovarian cancer.^90^ And tRF5^Glu^ could inhibit the proliferative ability of ovarian cancer by directly binding with the 3′UTR of BCAR3.^54^ Besides, tRNA^Gln^ was found to be upregulated in cervical cancer cells and inhibited protein translation, which was regulated by conserved residues in tRNA instead of combining target sites of mRNA.^91^ Collectively, tsRNAs can regulate cell proliferation by binding target genes, participating in pathways and performing other functions that are related to cell cycle, proliferation and migration, indicating tsRNAs have the potential to be detection biomarkers and (or) therapeutic targets; however, much more work needs to be performed to clarify the regulatory mechanisms and specific functional roles.

### 5.2 tsRNAs in metabolic diseases

It has long been understood that an individual's environment is not only influenced by their own metabolism but also increased the probability of their offspring developing metabolic diseases.^92^ For example, paternal preconception stress suffered in early life or adulthood is often conveyed to children. In addition, anxiety and depression‐related behaviours are part of hypothalamic‐pituitary‐adrenal axis dysregulation and can be relieved through running. Surprisingly, this phenomenon is accompanied by alterations in the expression levels of two tRFs, derived from tRNA^Gly^ and tRNA^Pro^. Moreover, this was the first evidence that the level of tsRNAs is related to the anxiolytic behavioural phenotype of male offspring.^93^ The results of this study were confirmed in a model of voluntary wheel running by mice, in which the control was a single‐compartment mouse with no opportunity for exercise, and each male rat from the experimental group was paired with a 10‐week‐old female rat and participated in a running wheel. The results showed that not only did the levels of tRNAs in the spermatozoa of the male mice in the treatment group change compared with the control group, but their offspring also showed lower levels of anxiety and reduced fear memory. A subsequent study showed that in mice fed a low protein diet, tRFGly‐GCC increased as spermatozoa matured in the epididymis, and genes associated with the endogenous retroelement MERVL were repressed not only in embryonic stem cells but also in embryos. Furthermore, tRFGly‐GCC was cleaved in the epididymis and then transported to spermatozoa to carry out its function, inhibiting MERVL‐regulated genes that affected placental size or function and then stimulating downstream effects on metabolism following altered placentation.^94^ Another study suggested that the offspring of mice fed a high‐fat diet (HFD) experienced the destruction of glucose tolerance and insulin secretion. Moreover, glucose tolerance was associated with sperm 5′tsRNAs. These tsRNAs contain numerous RNA modifications, such as 5‐methylcytidine (m5C) and N2‐methylguanosine (m2G), and the first offspring with metabolic disorders were created by influencing gene expression within metabolic pathways in the early embryo stage.^87^ As previously reported, intergenerational inheritance becomes possible because spermatozoa may obtain environmental information mediated by tsRNAs. These studies provide theoretical support for the regulatory function of tsRNAs in metabolic diseases and provide a new idea that metabolic diseases can reduce the hereditary rate of offspring by regulating the level of tsRNAs.

### 5.3 tsRNAs in neurological diseases

**FIGURE 4:**
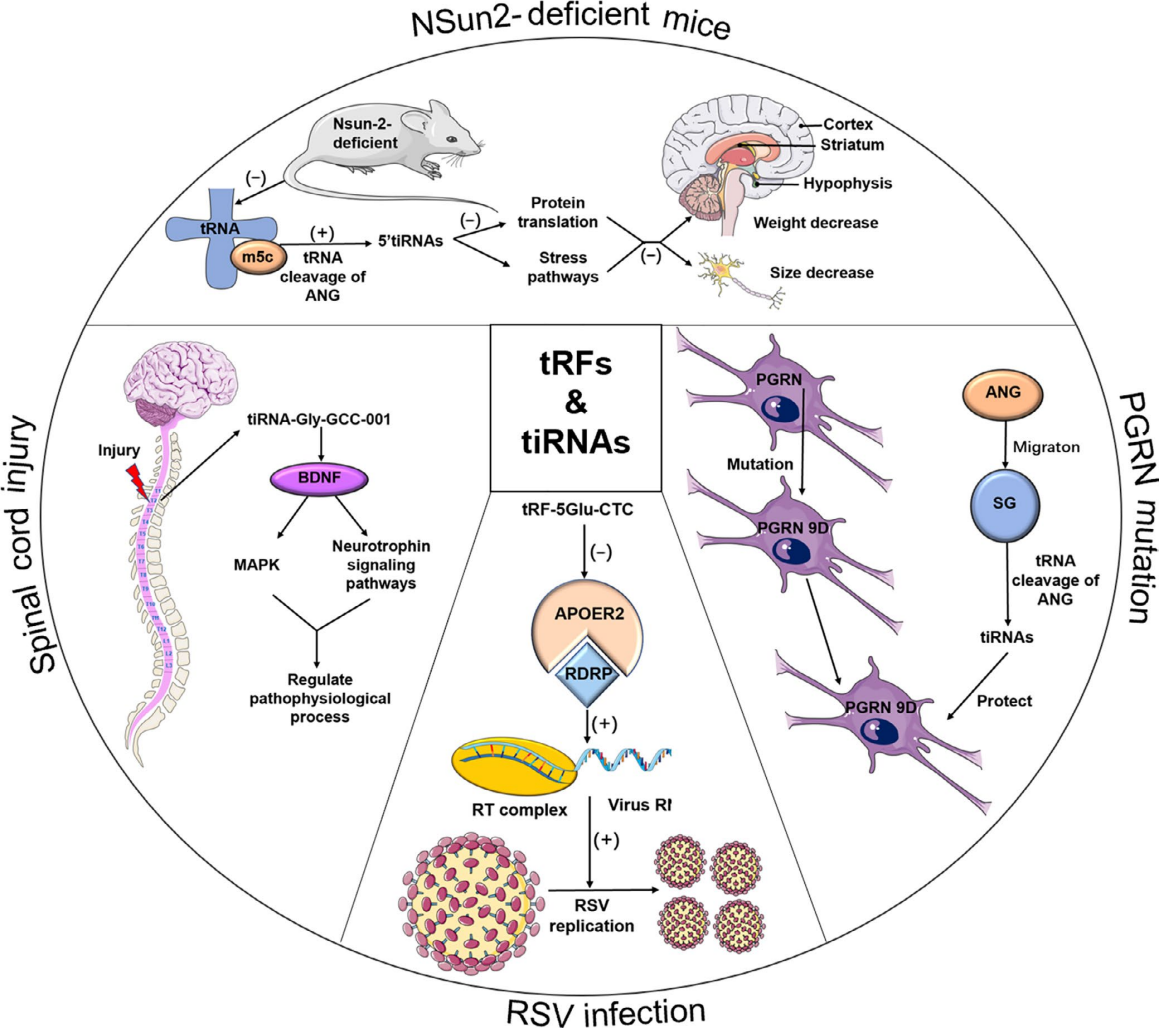
The role of tsRNA in various diseases. tsRNAs are widely involved in the progress of metabolic diseases, neurological diseases and viral infection diseases through regulating different functional mechanisms (see text for details)

Neurological diseases are diverse and complex, and of these, neurodegenerative diseases are characterized by a large loss of specific neurons, which deteriorate over time and eventually lead to dysfunction. In recent years, tsRNAs cleaved by ANG and ANG were found to be related to the survival or apoptosis of neurons in patients with neurodegenerative diseases, which indicated there are links between tsRNAs and the development of neurodegeneration^95^, ^96^ (Figure 4). A recent study found that the absence of cytosine‐5 RNA methylation, located at position 48 or 49 in tRNA, increased the ANG‐mediated cleavage of transfer RNAs in NSun2‐deficient mice, resulting in an accumulation of 5′tRNA‐derived small RNA fragments (tiRNAs). Further investigation showed NSun2^−/−^ cells were highly sensitive to cellular stress, and stability of tRNAs was reduced. Increasing tiRNAs slowed down protein synthesis and triggered stress responses, resulting in reduced size and weight of the brain, mainly in the cortex (Cx), hippocampus (Hp) and striatum (St) where NSun2 was highly expressed.^97^ Furthermore, the NSun2 mutation leads to some neurological diseases in humans, such as Dubowitz‐like syndrome and a syndrome form of intellectual disability.^98^, ^99^, ^100^ Additionally, ANG meditating the biogenesis of tiRNAs provides a clue that ANG may regulate the progression of neurological diseases. A mutation occurring in the hydrophobic core of the signal peptide in exon 1 of progranulin (PGRN) via the introduction of a charged amino acid, called PGRN A9D, has been recognized as an aetiology and activator of neurodegenerative diseases. However, PGRN A9D is distributed in cytoplasm but not the nucleus, which indicates a relationship with ANG‐meditated cellular distribution. PGRN A9D directed stress granule localization through ANG and then activated its cell‐protective stress response programme though tiRNAs.^101^ tsRNAs also appear in the prefrontal cortex, cerebrospinal fluid and serum in Parkinson patients with high sensitivity and specificity.^102^ A conclusion could be drawn that PGRN and ANG have common regulatory functions in the progression of neurodegenerative diseases, and tsRNAs are promising candidates that deserve further evaluation as non‐invasive clinical biomarkers. Apart from that, spinal cord injury (SCI) is another kind of neurological disease, usually caused by trauma. A recent study using tsRNA‐Seq analysis identified 297 differentially expressed tsRNAs obtained from rat spinal cord. Bioinformatic analyses and a luciferase reporter assay were used to show that tiRNA‐Gly‐GCC‐001 might exert a regulatory function via the mitogen‐activated protein kinase and neurotrophic signalling pathways by targeting brain‐derived neurotrophic factor.^103^ Additionally, we have reason to believe that tsRNAs have a potential therapeutic effect on neurodegenerative diseases caused by NSun2 deficiency and mutation, but more studies are still needed to confirm how to regulate tsRNA levels and the precise mechanism of action. Besides, in order to better study tsRNAs, many animal models and types are needed to verify that they can be used as potential detection indicators and therapeutic targets.

### 5.4 tsRNAs in viral infection diseases

Viruses invade the body in a variety of ways and proliferate in susceptible host cells, eventually causing diseases in a manner or via a mechanism that is not yet clear.^51^ Recently, tsRNAs were found to have a potential relationship with viral diseases. tsRNA abundance increases markedly in hepatitis B or hepatitis C and HBV‐ and HCV‐associated cancers, and this growth trend is consistent with ANG levels, suggesting there are specific HBV‐meditated mechanisms regulating tRNA biogenesis directly.^104^ Additionally, human respiratory syncytial virus (RSV), which often causes children to develop bronchiolitis and pneumonia, leads to abundant production of tsRNAs, especially tRF‐5^Glu‐CTC^ (Figure 4). As a target of tRF‐5^Glu‐CTC^, the gene expression of apolipoprotein E receptor 2 (APOER2) would be inhibited by the 3′‐portion of tRF‐5^Glu‐CTC^ recognizing and binding its 3′‐UTR. This inhibition benefits RSV replication because APOER2 is a kind of anti‐RSV protein.^51^, ^104^ A subsequent study also found that the quantity of tRF‐5 increases with rickettsia infection.^105^ tsRNAs have never been studied in human tissue before, so how does tsRNA abundance vary in different liver cell types, and how do tsRNAs function in liver cells? Research on tsRNAs is in an initial stage, and much work needs to be done to elucidate the tsRNA‐target network. Thus, this network has the potential to become a powerful theoretical source of tsRNAs as diagnostic and therapeutic factors for viral infections.

With the continuous development of sequencing technology, more and more tsRNAs differentially expressed in clinical samples of diseases have been discovered, including cancers, metabolic diseases, neurological diseases and viral infection diseases. Moreover, some studies even further explored the roles and mechanisms of several tsRNAs. These studies provide strong evidence that tsRNAs may be the promising diagnostic biomarkers and therapeutic targets.

## 6 DIAGNOSIS AND THERAPEUTIC PROPERTIES OF TSRNAS IN CLINICAL APPLICATION

### 6.1 sRNAs are potential ideal biomarkers for diagnosis and prognosis

At present, many clinical diseases lack specific diagnostic indicators, and most of them rely on pathological biopsy for diagnosis, which not only takes time but also poses invasive harm to patients. Especially for tumours, the lack of symptoms in the early stage causes the loss of the best time for treatment. When patients come to see a doctor due to symptoms, they are mostly in the late stage. Since cancer has not yet been conquered by human beings, most late‐stage cancers are treated with surgery, radiotherapy or chemical drugs. These long‐term treatments impose economic burden on patients and bring great physical and psychological pain to patients. Therefore, if diseases can be detected at an early stage through routine physical examination, it will be a great boon to patients. With the rapid development of science and technology, a large number of new detection technologies have emerged in different disease fields, among which tsRNAs have been gradually found to be involved in the process of cancer occurrence and development and have shown potential as biomarkers. Dramatically, their chemical properties are the most important advantages of tsRNAs as biomarkers. (a) Nucleotide modification, such as 5‐methylcytidine (m5C) and N2‐methylguanosine (m2G), increases the stability of tsRNAs. The levels of tsRNAs are substantially reduced without methylation, which indicates unmethylated tsRNAs are degraded by ribonucleases.^25^ This stability can also be sustained by binding a large‐size serum protein complex.^27^ The presence of such modifications greatly improves the stability of tsRNAs and lays an important foundation for their role as biomarkers. (b) tsRNAs are widely distributed in the body with high expression. The fraction of transcriptome‐aligned reads at >50% in biofluids indicates their abundance is very high. Additionally, they exist not only in tissues and cells but also in bile, urine, seminal plasma and amniotic fluid,^106^ which is very convenient for extractors given the reduced difficulty of detection. (c) tsRNAs are highly conserved in vertebrates. In the evolutionary tree, these serum tsRNAs (at least for tsRNA^Gly^ and tsRNA^Glu^) are ancient and small RNAs that maintain highly conserved sequences in vertebrates, such as fish, amphibians, reptiles, birds, mice, non‐human primates and humans.^27^ This trait is also a condition of a biomarker. (d) tsRNAs have unique expression patterns in different tissues and at different times. Tissue specificity and temporal specificity are two important expression patterns of tsRNAs, which are conducive to improving the specificity of tsRNA clinical detection, as well as differential diagnosis and prognosis.^27^ In conclusion, all of these properties indicate that tsRNAs have the potential to become specific and accurate biomarkers for diagnosing cancer and other diseases in different systems.

### 6.2 The potential advantageous therapeutics of tsRNAs

According to the study of tsRNAs in diseases, tsRNAs possess several advantages for applications in clinical therapy. (a) tsRNAs may act as direct therapeutic factors. Compared with a normal physiological state, the abundance of tsRNAs changes in a pathological state,^85^ which indicates tsRNAs may regulate the cell‐stimulating factor to promote or inhibit the occurrence and development of diseases. Despite the lack of numerous research, tsRNAs also might be critically involved in regulating the cell damage factors or cell‐protective factors, such as lysosomal enzyme^107^ or epidermal growth factor of gastric mucosa (EGF).^108^ And the level of tsRNAs can be regulated in different cells, local tissues or organs by regulating the physiological cleavage of tRNAs by ANG, the degradation rate of tsRNAs or via direct gene editing techniques. For example, tsRNAs can be injected intravenously to increase the level of tsRNAs, while injection of ANG may reduce the level of tsRNAs in the body. Even with the advancement of technology in genetic modification and editing, tsRNAs can be directly encoded and synthesized in the body. These various approaches can regulate the level of tsRNAs and directly regulate the progression of the disease, avoiding the side effects of conventional treatments. (b) tsRNAs serve as indirect therapeutic factors. In addition to exerting direct functions, tsRNAs can also play an indirect role in regulating disease progression. First, there are many signalling pathways in the process of disease progression. tsRNAs can bind to certain targets in the signalling pathway,^85^ activate or inactivate the binding targets to start or stop signal transmission, and then exert a therapeutic effect. For example, 5′‐tiRNA^Val^ can inhibit FZD3/Wnt/β‐Catenin signalling in breast cancer by binding with FZD3.^85^ Second, tsRNAs regulate the cell cycle to accelerate or inhibit cell proliferation, which in turn can be used to repair damage and inhibit cancer. For example, tRF‐1001 suppressed cell proliferation by arresting cancer cells at the G2 phase. So, it is supposed the level of tsRNAs with protective effect might be increased by intravenous injection of which while tsRNAs with pathogenic effect might be decreased by activating ribonucleases to cleave maternal gene. With the development of science and technology, more methods will be found to regulate the level of tsRNAs.

tRNA and tsRNAs are abundant in biological fluids, sometimes even higher than miRNA.^109^, ^110^ In addition, although the current screening of body fluid biomarkers is mainly focused on miRNA, tsRNAs show several advantages in a variety of body fluids. For example, partial least squares discrimination analysis (PLS‐DA) analysis found that the expression profile of tsRNAs can clearly distinguish negative (or positive) breast cancer from normal controls^111^; the study also shows that the proportional relationship between different tsRNAs can be used as an efficient cancer progression‐free survival (PFS) indicators and diagnostic marker candidates.^109^ These have opened up broad prospects for tsRNAs to become low‐invasive biomarkers. In addition, tsRNAs are derived from tRNA, so they carry modifications that make themselves more stable. Compared with miRNAs, they are more stable in body fluids, blood and cells.^89^ Moreover, miRNA plays the function of silencing downstream targets mainly by binding to AGO2. In addition to having similar functions to miRNAs, tsRNAs can also bind to AGO1, AGO3, AGO4 or regulate RNA silencing in an AGO‐independent manner.^76^ This finding suggests that tsRNAs can regulate RNA in more ways, providing a broader therapeutic prospect for tsRNAs. Since tsRNAs themself have many modifications, conventional small RNA detection methods cannot be applied. At present, the tsRNAs pre‐treatment kit has been developed, which can effectively remove the 3′aminoacyl end, 3′cyclic phosphate end, phosphorylated 5′hydroxyl end and various methylation modifications such as m1A and m3C. In addition, tsRNAs are derived from tRNAs and are highly modified, so the function of specific tsRNAs may be closely related to their modification, but this is largely unproven.^112^ Currently, the research on the molecular functions of tsRNAs is almost all artificially synthesized RNA sequences. This modification rarely contains modifications, so it is very likely that the effect of RNA modification on the function of tsRNA is neglected.^113^ For example, when studying the epigenetic information carried by the modification of tsRNAs, extracted tsRNAs are used, while artificial unmodified RNA molecules do not perform epigenetic functions.^57^ Theoretically, once the mode and location of the modification are determined, the expert laboratory can synthesize the modified nucleotides introduced at the specific location.^112^ However, the current commercial use still needs further research and design.

### 6.3 tsRNA sequencing analysis in patient samples

Based on the above analysis, since tsRNAs have the potential for diagnosis and prognosis, many studies have also been carried out in clinical samples to find tsRNA biomarkers that can be applied in clinical practice (Table 3). A study of breast cancer using high‐throughput sequencing technology found that 30 tRFs and tiRNAs were differentially expressed in six pairs of breast cancer patients and self‐matching adjacent non‐tumour breast tissues, of which 17 were upregulated and 13 were downregulated. Next, to further verify the accuracy of sequencing data, the study collected and tested the expression levels of tRFs and tiRNAs in 16 breast cancer tissue samples. Interestingly, the expression level of AS‐tDR‐001430 in tumour samples was significantly lower than that in non‐tumour samples, and this result was consistent in serum samples from 60 patients and in the corresponding healthy control group. And the sensitivity and specificity for diagnosing cancer and normal tissues are 90.0%, and the specificity is 62.7%. At the same time, AS‐tDR‐001430 was found to be negatively correlated with the TNM staging of tumour tissue and was significantly correlated with lymph node metastasis.^85^ For example, the sensitivity and specificity for diagnosing early and advanced cancer are 85.0% and 90.0%, 51.9% and 75.9%, respectively. The sensitivity for diagnosing distant lymph node metastasis is 89.0%, and specificity is 70.6%. Therefore, we had reason to believe that AS‐tDR‐001430 could be used not only as a diagnostic indicator for breast cancer staging but also as a tumour inhibitor to suppress the progression of breast cancer. In addition, 86 gastric cancer tissues and adjacent paired non‐tumour tissues were collected, and the expression level of tiRNA5034‐GluTTC‐2 was observed to be significantly lower in gastric cancer tissues.^89^ Then, paired plasma samples were collected from gastric cancer patients 1 day before and 7 days after surgery. The results showed that the levels of tiRNA5034‐GluTTC‐2 before and after surgery were significantly downregulated compared with those in the healthy group. More importantly, multivariate analysis of the study data showed the potential value of tiRNA5034‐GluTTC‐2 in the diagnosis of gastric cancer. Apart from these tsRNAs above, several tsRNAs were found dysregulated in CLL blood and EC tissue.^86^, ^114^

**TABLE 3:** tsRNAs as potential biomarkers identified in clinical samples

tsRNAs	Expression pattern	Fold change (*P* < .05)	Samples	Ref.
AS‐tDR‐001328	Upregulated	2.6	Breast tissue	85
AS‐tDR‐000779	Upregulated	2.6		
AS‐tDR‐001443	Downregulated	2.0		
AS‐tDR‐000693	Downregulated	2.0		
tiRNA‐5034‐GluTTC‐2	Downregulated	0.733	Gastric tissue and plasma	89
ts‐4521	Downregulated	3‐8	CLL blood	114
tDR‐000894	Upregulated	2.95	EC tissue	86
tDR‐001307	Upregulated	1.88		
tDR‐001361	Upregulated	2.4		
tDR‐001292	Downregulated	4.1		

These clinical studies show that, whether in tissue samples or in plasma samples, tsRNAs are significantly differentially expressed in diseases and control groups. Taken together, these results indicate that tsRNAs exhibit significant advantages in disease detection and (or) therapeutic treatment.

### 6.4 tsRNAs exist in microvesicles and exosomes

**FIGURE 5:**
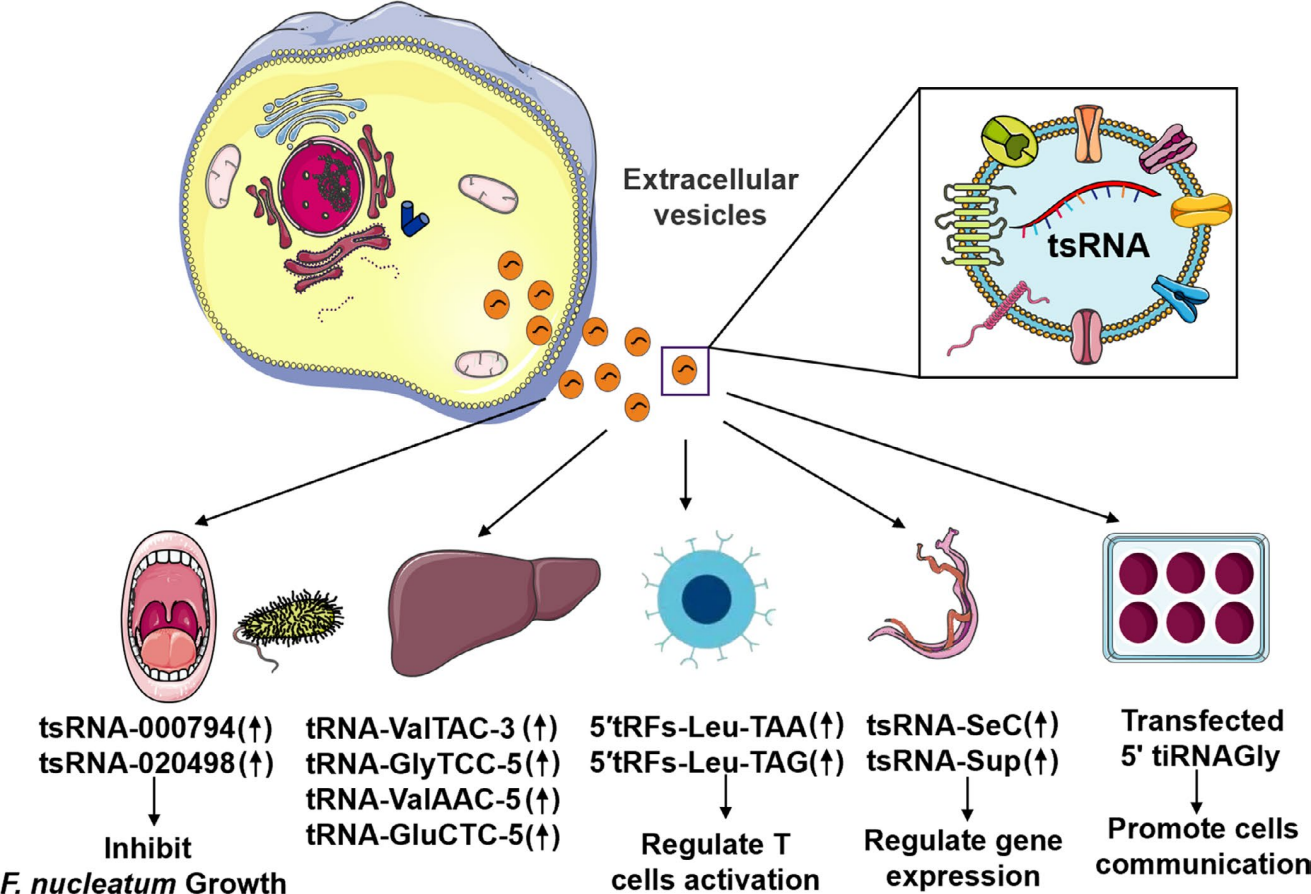
A schematic representation of exosomal tsRNAs in regulating a variety of cells and diseases. tsRNAs could inhibit the growth of *Fusobacterium nucleatum* in the oral cavity. In addition, tsRNAs were significantly increased in exosomes isolated from the blood of liver cancer patients. tRFs in EVs had the potential to regulate immune response by affecting T‐cell activation. Exosomal tsRNAs released by *Schistosoma mansoni* could regulate gene expression of eukaryotic cells. Besides, 5ʹ tiRNA‐Gly can convey stress‐related signals between adjacent MCF‐7 cells

Exosomes are exocytic microvesicles (30‐100 nm) released by healthy and pathological cells, which carry DNAs, RNAs and proteins to promote communication between different cells which are generally considered as promising therapeutics in future.^17^, ^115^ Studies had already found several ncRNAs, such as miRNAs and lncRNAs, are abundant in exosomes and interact with recipient cells to regulate cell function and disease progresses.^116^, ^117^, ^118^ Likewise, recent studies showed tsRNAs are widely exist in microvesicles and exosomes, which could protect themselves from degradation and make them stable components of body fluids.^88^ What's more, current studies had also confirmed that tsRNAs could transfer from transfected cells to recipient cells through exosomes,^119^ and play roles by this special delivery system (Figure 5). For example, *Fusobacterium nucleatum*, a kind of Gram‐negative oral bacteria, could stimulate the release of tsRNA‐000794 and tsRNA‐020498 embedded in exosomes in human normal oral keratinocyte cells. In reverse, these two tsRNAs inhibited the growth of *F nucleatum* by disturbing protein synthesis.^120^ Moreover, further study verified tRFs in EVs had the potential to regulate immune response by affecting T‐cell activation.^88^ Surprisingly, exosomal tsRNAs could be released by *Schistosoma mansoni* to regulate gene expression in eukaryotic cells.^121^ Especially, the study found that the expression levels of four tsRNAs (tRNA‐ValTAC‐3, tRNA‐GlyTCC‐5, tRNA‐ValAAC‐5 and tRNA‐GluCTC‐5) were significantly increased in exosomes extracted from the blood of patients with liver cancer compared with the control group,^122^ implying they had the potential of becoming diagnostic biomarkers. However, there is no unified exosome separation method, and the low purity of exosomes is one of the unsolved problems. In addition, the isolation process of exosomes may break up and reduce the concentration of non‐coding RNA.^123^, ^124^ In order to remove the contents of dead cells and non‐vesicle, recent study used two‐step ultracentrifugation method to isolate EVs from T cells.^88^ Although this separation method improved the purity of exosomes, more effective strategies are absolutely needed for further investigation. To sum up, tsRNAs in exosomes can increase their stability and make them potential detection biomarkers as well as minimal invasive therapeutic tools.

## 7 CONCLUSIONS AND PROSPECTS

Due to the continuous development and application of sequencing analysis technology, more and more tsRNAs are gradually being discovered. Moreover, their function and mechanisms in diseases have been continuously studied, enriching our understanding of tsRNAs. However, several limitations are still need to be solved. First, we do not fully understand the biological characteristics and mechanisms of tsRNAs. There are many types of tsRNAs, and our current knowledge of tsRNAs is mainly limited to a small number of tsRNAs. Therefore, it remains unclear whether their expression level, distribution or biological function is universal. Second, there are no formal rules for naming tsRNAs. Currently, the primary classification is two major subtypes, tRFs and tiRNAs, but the naming of the lower classification is still confusing which need further standardization. In some literature sequencing results, tsRNAs are called tDRs; for example, AS‐tDR‐001430 is called instead of 5‐tiRNA^Val^.^85^ tRF^Ser‐TGA^ is called Cand45,^30^ tRF^Glu^ is called td‐piR (Glu).^125^ And currently, there are other names for tiRNA, such as tRNA halve and SHOT‐RNA.^39^, ^126^ Third, only a small fraction of the tsRNAs in the sequencing map meet the criteria for biomarkers. Although tsRNAs show high specificity and sensitivity, the methods for tsRNA detection are mainly high‐throughput sequencing, which are limited in the analysis of large clinical samples. Their potential applications in diagnosis, prognosis and treatment in clinical trials have rarely been demonstrated. Fourth, the origins and whereabouts of tsRNAs remain unclear. Although RNase Z, Dicer and ANG are known to produce tsRNAs by cleaving specific sites of tRNAs, the ribonuclease responsible for 2‐tRF biogenesis has not yet been discovered, nor is it known if there is any possibility of other patterns of cleavage or post‐transcriptional modification. Finally, the tools used to identify tsRNAs and their targets are still in their infancy. The Kyoto Encyclopaedia of Genes and Genomes (KEGG) Pathway and Gene Ontology (GO) Biological Processes are always used to indicate the biological functions and pathway analyses of differentially expressed tRFs/tiRNAs.^86^ The bioinformatic analysis of predicting downstream targets is not yet complete, which hinders the study of tsRNAs. Therefore, tsRNA research needs to overcome these limitations in order to achieve long‐term development.

Although at present our research on tsRNAs is still in its infancy, the expression levels of tsRNAs in physiological and pathological conditions are obviously different, and we have reason to believe that tsRNAs have very good research prospects in cancers, metabolic diseases, neurodegenerative diseases, viral infections and additional diseases. First, tsRNA can be used as a biomarker for disease occurrence, development and prognosis. For example, a study on gastric cancer showed that tiRNA5034‐GluTTC‐2 may be a new biomarker because the expression level of tiRNA‐5034‐GluTTC‐2 is negatively correlated with the size of the tumour. In addition, in the survival curve, the overall survival time of patients in the high expression group of tiRNA5034‐GluTTC‐2 was significantly higher than that of the low expression group, suggesting that tiRNA5034‐GluTTC‐2 is an independent predictor of gastric cancer prognosis.^89^ Second, tsRNAs may be used as a means of clinical treatment target. It is reported that extracting tsRNAs from spermatozoa of HFD male mice and injecting it into the fertilized eggs of normal mice will cause metabolic disorders in the F1 generation offspring and changes in the expression of genes related to islet metabolism. Suppose that in order to block the occurrence of paternal inherited metabolic diseases, we may achieve this by promoting tsRNA degradation or inhibiting tsRNA production.^87^ In addition, tsRNAs can be used in clinical therapy, not only as direct therapeutic targets but also to regulate disease progression through indirect regulation of signalling pathways. By regulating the expression of tsRNAs, blocking or delaying the occurrence and development of diseases can be a suitable clinical application strategy for tsRNAs. It is worth mentioning that tsRNAs are abundantly expressed, widely distributed in the body and modified to improve stability. These characteristics are strong evidence that tsRNAs have the potential to become non‐invasive diagnostic or therapeutic methods.^89^ With the development of science and technology, more new technologies will emerge and see constant improvement, which will be conducive to the identification of more tsRNAs that can be developed further into clinically available biomarkers and therapeutic targets, providing a new direction and perspective for the diagnosis and treatment of diseases.

## CONFLICT OF INTEREST

The authors have declared no conflicting interests.

## AUTHOR CONTRIBUTIONS

ZT, FX, LL and WZ collected articles and designed the manuscript. ZT, YY and ZH wrote the manuscript. TG and LM prepared tables and figures. YT, ALHH, ZH, LP and WJ revised and approved the manuscript. All authors read the manuscript and approved the final manuscript.

## Data Availability

Data sharing is not applicable to this article, so no new data were created or analysed in this study.
